# Retinoic acid inducible gene-I mediated detection of bacterial nucleic acids in human microglial cells

**DOI:** 10.1186/s12974-020-01817-1

**Published:** 2020-05-01

**Authors:** M. Brittany Johnson, Justin R. Halman, Amanda R. Burmeister, Saralynn Currin, Emil F. Khisamutdinov, Kirill A. Afonin, Ian Marriott

**Affiliations:** 1 Department of Biological Sciences, University of North Carolina at Charlotte, 9201 University City Blvd, Charlotte, NC 28223 USA; 2 Nanoscale Science Program, Department of Chemistry, University of North Carolina at Charlotte, Charlotte, NC 28223 USA; 3 Center for Neurodegenerative Science, Van Andel Institute, Grand Rapids, MI 49503 USA; 4 Department of Chemistry, Ball State University, Muncie, IN 47306 USA

**Keywords:** RIG-I, Human, Microglia, Bacterial nucleic acids, Nucleic acid nanoparticle

## Abstract

**Background:**

Bacterial meningitis and meningoencephalitis are associated with devastating neuroinflammation. We and others have demonstrated the importance of glial cells in the initiation of immune responses to pathogens invading the central nervous system (CNS). These cells use a variety of pattern recognition receptors (PRRs) to identify common pathogen motifs and the cytosolic sensor retinoic acid inducible gene-1 (RIG-I) is known to serve as a viral PRR and initiator of interferon (IFN) responses. Intriguingly, recent evidence indicates that RIG-I also has an important role in the detection of bacterial nucleic acids, but such a role has not been investigated in glia.

**Methods:**

In this study, we have assessed whether primary or immortalized human and murine glia express RIG-I either constitutively or following stimulation with bacteria or their products by immunoblot analysis. We have used capture ELISAs and immunoblot analysis to assess human microglial interferon regulatory factor 3 (IRF3) activation and IFN production elicited by bacterial nucleic acids and novel engineered nucleic acid nanoparticles. Furthermore, we have utilized a pharmacological inhibitor of RIG-I signaling and siRNA-mediated knockdown approaches to assess the relative importance of RIG-I in such responses.

**Results:**

We demonstrate that RIG-I is constitutively expressed by human and murine microglia and astrocytes, and is elevated following bacterial infection in a pathogen and cell type-specific manner. Additionally, surface and cytosolic PRR ligands are also sufficient to enhance RIG-I expression. Importantly, our data demonstrate that bacterial RNA and DNA both trigger RIG-I-dependent IRF3 phosphorylation and subsequent type I IFN production in human microglia. This ability has been confirmed using our nucleic acid nanoparticles where we demonstrate that both RNA- and DNA-based nanoparticles can stimulate RIG-I-dependent IFN responses in these cells.

**Conclusions:**

The constitutive and bacteria-induced expression of RIG-I by human glia and its ability to mediate IFN responses to bacterial RNA and DNA and nucleic acid nanoparticles raises the intriguing possibility that RIG-I may be a potential target for therapeutic intervention during bacterial infections of the CNS, and that the use of engineered nucleic acid nanoparticles that engage this sensor might be a method to achieve this goal.

## Introduction

Bacterial meningitis and meningoencephalitis are serious conditions that result in permanent neurological deficits and even death. It is now appreciated that resident glia, such as microglia and astrocytes, identify conserved pathogen motifs via pattern recognition receptors (PRR), thereby triggering both protective and detrimental immune responses to bacterial infection of the central nervous system (CNS) [1–5]. Glial cells express a variety of PRRs, including members of the Toll-like receptor (TLRs), nucleotide oligomerization domain (NOD)-like receptor (NLR), and retinoic acid inducible genes (RIG)-I-like receptor (RLRs) families [6–11]. TLRs have been extensively characterized and are known to detect extracellular and endosomal pathogen motifs to trigger inflammation [6, 12, 13]. In contrast, the cytosolic PRRs such as the NLRs and RLRs have been identified relatively recently, and the role of these sensors in glial cell responses to bacterial infection remains poorly understood.

The importance of RLRs as viral PRRs in glial cells is well established [3, 14–17]. RIG-I binding to cytosolic RNA ligands that contain 5′-triphosphate groups triggers IRF3 phosphorylation, dimerization, and nuclear translocation, leading to the induction of interferons (IFN) and cytokine production [18–24]. Type I IFNs that are produced following RIG-I activation can act in an autocrine or paracrine manner to stimulate expression of IFN-stimulated genes (ISGs) that sensitize a cell for pathogen detection by upregulating PRR expression and stimulating immune mediator release [25]. In glial cells, we have demonstrated that RIG-I is necessary for viral recognition and stimulation of immune responses to the neurotropic RNA virus, vesicular stomatitis virus (VSV), in murine microglia and primary human astrocytes [3, 9]. Additionally, we have previously shown that RIG-I contributes to maximal murine glial cell responses to the DNA virus, herpes simplex virus-1 (HSV-1), in an RNA polymerase III-dependent manner [14].

Intriguingly, recent evidence indicates that RIG-I may also play a novel role in the detection of bacterial pathogens. RIG-I has been demonstrated to identify *Legionella pneumophila*, *Shigella flexneria*, *Listeria monocytogenes*, and *Salmonella enterica serovar Typhimurium* in peripheral cell types [22, 26–32]. Interestingly, these studies suggest that RIG-I identification of cytosolic bacterial RNA or DNA is pathogen dependent. For example, RIG-I appears to recognize *Shigella flexneria* DNA indirectly via the action of RNA polymerase III, but this cytosolic sensor can detect both RNA and DNA of *L*. *monocytogenes* and *L*. *pneumophila* [22, 27, 29, 30]. Furthermore, there is evidence to suggest that RIG-I identification of bacterial RNA versus DNA is also cell type-dependent, as RIG-I-dependent production of IFN is only observed following *S*. *enterica serovar Typhimurium* infection of non-phagocytic cells [28]. Similarly, *L*. *monocytogenes* directly stimulates RIG-I -mediated recognition of RNA in human monocytes, epithelial cells, and hepatocytes, but exclusively mediates recognition of DNA in human monocytes [30–32]. Together, these data indicate the particular pathogen and host cell type in combination determine the role of RIG-I in pathogen identification. To date, the importance of RIG-I in the detection of bacteria by human glial cells has not been determined.

In the present study, we demonstrate that RIG-I is constitutively expressed by human glial cells and show that such expression is further upregulated in response to bacterial infection or exposure to bacterial products that serve as ligands for surface and cytosolic PRRs. Importantly, we show that bacterial RNA and DNA both trigger RIG-I-dependent IRF3 phosphorylation and subsequent type I IFN production in human microglia. This ability was confirmed in studies using novel engineered nucleic acid-based nanoparticles (NANPs) [33–35] where we demonstrate that both RNA- and DNA-based nanoparticles can stimulate RIG-I-dependent IFN responses in human microglial cells. As such, RIG-I may be a potential target for therapeutic intervention during bacterial infections of the CNS, and the use of engineered NANPs that engage this sensor might be a method to achieve this goal.

## Materials and methods

### Source and propagation of human glial primary cells and cell lines

Primary human astrocytes were purchased from ScienCell Research Laboratories (Carlsbad, CA). These cells were isolated from human cerebral cortex, characterized by the vendor by immunofluorescence for glial fibrillary acidic protein (GFAP), and cryopreserved at passage one. The immortalized human astrocytic cell line, U87-MG, was obtained from the American Type Culture Collection (ATCC; HTB-14). Cells were maintained in Eagle minimum essential media (EMEM) supplemented with 10% fetal bovine serum (FBS) and 100 U/ml penicillin–100 μg/ml streptomycin at 37 °C 5% CO_2_. A human microglia cell line (hμglia) was a generous gift from Dr. Jonathan Karn (Case Western Reserve University). These cells were derived from primary human cells transformed with lentiviral vectors expressing SV40 T antigen and human telomerase reverse transcriptase. The characterization and classification of this cell line has been previously described [10, 36, 37]. These cells are classified as microglia due to microglia-like morphology, expression of the microglia surface markers CD11B, TGFβR, and P_2_RY_12_, and their migratory and phagocytic activity. Cell were maintained in Dulbecco’s modified Eagle’s medium (DMEM) supplemented with 5% FBS and 100 U/ml penicillin–100 μg/ml streptomycin at 37 °C 5% CO_2._

### Murine glial cell isolation and culture

Primary murine glial cells were isolated as described previously by our laboratory [1, 6, 8, 38, 39]. Briefly, six to eight neonatal C57BL/6J mouse brains per preparation were dissected free of meninges and large blood vessels, minced using sterile surgical scissors, incubated with 0.25% trypsin 1 mM EDTA in serum-free RPMI 1640 medium for 5 min, and forced through a wire screen. The cell suspension was pelleted and suspended in RPMI 1640 containing 10% FBS and penicillin-streptomycin mix for 2 weeks. Astrocytes were isolated from mixed glial cultures by trypsinization (0.25% trypsin–1 mM EDTA for 20 min) in the absence of FBS [40]. The remaining intact layer of adherent cells was demonstrated to be > 98% microglia via immuno-histochemical staining for the microglial surface marker CD11b [40]. Isolated astrocytes were determined to be > 96% pure based on morphological characteristics and the expression of the astrocyte marker GFAP as determined by immunofluorescence microscopy [6]. Microglia were maintained in RPMI 1640 with 10% FBS and 20% conditioned medium from LADMAC cells (ATCC number CRL-2420), a murine monocyte-like cell line that secretes colony-stimulating factor-1 (CSF-1), for 24 h prior to experiments. It is important to note that prior to separation of mixed glial cultures, astrocytes produce the CSF-1 necessary to maintain microglial cells. Post separation, microglia were cultured in media containing the 20% conditioned medium from LADMAC cells to provide the necessary CSF-1. Astrocytes were maintained in RPMI 1640 containing 10% FBS for 24 h prior to experiments. All studies were performed in accordance with relevant federal guidelines and institutional policies regarding the use of animals for research purposes.

### Bacterial propagation

*Neisseria meningitidis* strain MC58 (ATCC BAA-335) was grown on Columbia agar plates supplemented with 5% defibrinated sheep blood (BD, Franklin Lakes, NJ) and cultured in Columbia broth (BD Biosciences, San Jose, CA) on an orbital rocker at 37 °C with 5% CO_2_ overnight prior to in vitro challenge. *Streptococcus pneumoniae* strain CDC CS109 (ATCC 51915) and *Salmonella enterica serovar Typhimurium* SB300 (provided by Dr. Michael C. Hudson, formally of the University of North Carolina at Charlotte) were grown from frozen stock on commercially available trypticase soy agar with 5% sheep blood (BD Biosciences). *Staphylococcus aureus* strain UAMS-1 (ATCC 49230) was grown on lysogeny broth (LB) agar plates. *S*. *pneumoniae*, *S*. *aureus*, and *S*. *typhimurium* were cultured overnight in tryptic soy broth on an orbital rocker at 37 °C with 5% CO_2_ overnight prior to in vitro challenge. The number of colony forming units (CFU) for each bacterial species was determined by spectrophotometry using a Genespec3 spectrophotometer (MiraiBio Inc., Alameda CA). Bacterial DNA and RNA were isolated using the commercially available kits, GeneElute bacterial genomic DNA, and RNeasy protect bacteria mini kit (Sigma and QIAGEN).

### Bacterial infection

Glial cells were infected with bacteria at multiplicities of infection (MOI) of 1, 10, or 50 bacteria to glia in antibiotic-free medium for 2 h at 37 °C with 5% CO_2_. These doses are based on bacterial numbers previously reported in the cerebral spinal fluid of children with bacterial meningitis [41]. After 2 h of infection, media containing penicillin-streptomycin (MilliporeSigma, St. Louis, MO) was added to kill extracellular bacteria. At the indicated time points following challenge, cell supernatants, whole cell protein lysates, and RNA were isolated for ELISAs, immunoblot analysis, and RT-PCR, respectively.

### Nuclear translocation

At the indicated time points, hμglia cells were suspended in a pH 7.9 lysis buffer containing 10 mM HEPES, 1.5 mM MgCl_2_, 10 mM KCl, 0.5 mM DTT, 0.05% NP40, and protease inhibitor cocktail for 10 min at 4 °C. The nuclei and other fragments were pelleted by centrifugation and supernatants were retained as cytoplasmic fractions. Nuclei were lysed by exposure to pH 7.9 high salt buffer containing 5 mM HEPES, 1.5 mM MgCl_2_, 0.2 mM EDTA, 0.5 mM DTT, 26% glycerol, and 300 mM NaCl for 30 min at 4 °C. Samples were cleared of cellular debris by centrifugation, and supernatants containing the nuclear fraction were subjected to immunoblot analysis using a rabbit monoclonal antibody specific for total IRF-3 (Cell signaling).

### Immunoblot analysis

Cell lysates were evaluated for the presence of RIG-I, RNA polymerase III subunit A, and phosphorylated IRF3 (pIRF3) by immunoblot analyses [9]. Blots were incubated with a rabbit polyclonal antibody against mouse and human RIG-I (Abgent, cat# AP1900a, 0.5 μg/ml), a rabbit monoclonal antibody specific for RNA polymerase III subunit A (Cell Signaling, cat # 12825S, 1:1000), a rabbit monoclonal antibody specific for IRF-3 phosphorylated at Ser396 (Cell Signaling, cat# 4947, 1:1000), or a rabbit monoclonal antibody for total IRF-3 (Cell Signaling, cat# 4302, 1:1000) overnight at 4 °C. Blots were then washed and incubated in the presence of a horseradish peroxidase (HRP)-conjugated secondary anti-rabbit antibody. Bound antibody was detected with WesternBright ECL kit (Advansta). Immunoblots were reprobed with a mouse monoclonal antibody against β-actin (Abcam, cat# 49900, 0.13 μg/ml) to assess total protein loading. Immunoblots shown are representative of at least three separate experiments and ImageLab software (BioRad) was used for densitometric analysis.

### Quantification of cytokines in glial cell supernatants

To quantify human IL-6 and IFN-β production, specific capture ELISAs were performed. A rat anti-human IL-6 capture antibody (BD Pharmingen, cat# 554543; Clone Mq2-13A5, 2 μg/ml) and a biotinylated rat anti-human IL-6 detection antibody (BD Pharmingen, cat# 554546; Clone MQ2-39C3, 2 μg/ml) were used in IL-6 ELISAs. While, a polyclonal rabbit anti-human IFN-β capture antibody (Abcam, cat# ab186669, 0.25 μg/ml) and a biotinylated polyclonal rabbit anti-human IFN-β detection antibody (Abcam, cat# ab84258, 0.25 μg/ml) were used in IFN-β ELISAs. Bound antibody was detected using streptavidin-HRP (BD Biosciences) followed by the addition of tetramethylbenzidine substrate. H_2_SO_4_ was used to stop the reaction and absorbance was measured at 450 nm. A standard curve was generated using dilution of recombinant cytokines for IL-6 (BD Pharmingen) and IFN-β (Abcam). The cytokine concentration in cell supernatants was determined by extrapolation of absorbance to the standard curve.

### Ligand stimulation

Glial cells were exposed to bacterial lipopolysaccharide (LPS) isolated from *Escherichia coli* (MilliporeSigma), Pam3Cys-Ser-(Lys)4 (Pam3Cys; InvivoGen, San Diego, CA), bacterial flagellin isolated from *Salmonella typhimurium strain* 14028 (Enzolife Sciences, Farmingdale, NY), or polyinosinic polycytidylic acid (polyI:C; MilliporeSigma). Additionally, glial cells were transfected with 5′ppp RNA (Invivogen), BDNA (dA:dT) (Invivogen), or RNA/DNA isolated from *Neisseria meningitidis* strain MC58 (ATCC BAA-335), *Streptococcus pneumoniae* strain CDC CS109 (ATCC 51915), and *Staphylococcus aureus* strain UAMS-1 (ATCC 49230) using an RNA isolation kit or genomic DNA isolation kit. Genomic DNA isolation included RNase treatment to remove contaminating RNA. Any potential DNA contamination was removed from isolated bacterial RNA using a DNase I kit (Sigma-Aldrich) and we confirmed that the hμglia human microglial cell line produces significant levels of IFN-β in response to transfection with DNase-treated *N*. *meningitidis* or *S*. *aureus* RNA (556 pg/ml and 679 pg/ml respectively).

### Transfection

Transfection of hμglia cells was conducted using lipofectamine 2000 (L2K, Invitrogen) according to the manufacturer’s guidelines. Ligands were incubated for 30 min with lipofectamine 2000 prior to transfection of hμglia with 0.1 μg/ml BDNA, 1 μg/ml 5′pppRNA, 0.5 μg/ml bacterial gDNA, 1 μg/ml bacterial RNA, or 5 nM nucleic acid nanoparticles for 4 h in DMEM supplement with 5% FBS. Cell culture media was subsequently changed to media additionally supplemented with 100 U/ml penicillin–100 μg/ml streptomycin at 4 h post transfection. Cell lysates and supernatants were collected for analysis at the indicated time points.

### BX795 treatment

In some experiments, microglia were untreated or treated with 1 μM BX795 (Invivogen) in DMEM supplemented with 5% FBS and 100 U/ml penicillin–100 μg/ml streptomycin at 37 °C 5% CO_2_ for 3 h prior to transfection with bacterial nucleic acids or nucleic acid nanoparticles as described above. BX795 blocks the phosphorylation of TANK-binding kinase 1 (TBK1)/IkappaB kinase-ε (IKKε) which inhibits the catalytic activity of these proteins. These signaling molecules are downstream of RIG-I ligand binding and are required for IRF3 phosphorylation and nuclear translocation. Cell lysates and supernatants were collected for analysis at the indicated time points.

### siRNA knockdown

Microglia were transfected with 5 nM control siRNA (silencer select negative control number 1 siRNA ThermoFisher Scientific), siRNA targeted against RIG-I (αRIG-I) (ThermoFisher Scientific assay identification number s223615), or siRNA targeting RNA polymerase III subunit A (Thermo Fisher Scientific assay identification number s21945), 48 h prior to transfection with bacterial nucleic acids or nucleic acid nanoparticles as described above. Silencer select siRNA was transfected according to the manufacture’s guidelines using RNAimax (ThermoFisher Scientific). Cells were placed in fresh media for 24 h prior to transfection with bacterial nucleic acids or nucleic acid nanoparticles. Cell lysates and supernatants were collected for analysis at the indicated time points.

### Nucleic acid-based nanoparticles assembly

All individual DNA oligonucleotides were purchased from Integrated DNA technologies and dissolved in Hyclone HyPure water cell culture grade (GE Healthcare Life Sciences). RNA strands were synthesized by run-off transcription of PCR-amplified DNA templates carrying the T7 RNA polymerase promoter region and amplified DNA products were subjected to an in vitro transcription with T7 RNA polymerase [42]. Transcribed RNAs were purified by denaturing gel electrophoresis (8% acrylamide, 29:1 acrylamide:bis-acrylamide, 8 M urea) and extracted from excised gel slices using 0.5 ml of a buffer containing 89 mM tris-borate, pH 8.2, 1 mM EDTA, 0.3 M of sodium chloride, with overnight shaking at 4 °C. RNAs were ethanol precipitated (3:1 volume ratio), rinsed twice with cold 90% ethanol, dried, and re-dissolved in molecular grade water. To assemble RNA and DNA triangles, individual strands were mixed at equimolar concentrations (at 1 or 5 μM final) in assembly buffer (89 mM tris-borate pH = 8.2, 2 mM MgCl_2_, 50 mM KCl), heated to 80 °C for 5 min, and slow cooled to 4 °C over a 1-h period (Supplemental 1). The assembly of triangles was confirmed by 6% native-PAGE with subsequent staining with ethidium bromide and visualization using the BioRad Gel Doc system.

### Atomic force microscopy imaging

Thirty microliters of assembled triangles at a concentration of 5 μM were subjected to an 8% non-denaturing PAGE. The gel was run for 50 min at a constant voltage of 90 and triangles were visualized using UV shadowing, then cut, and eluted from the gel using 500 μl of assembly buffer (89 mM tris-borate pH = 8.0, 2 mM MgCl_2_, 50 mM KCl) overnight. Triangles were then precipitated using three volumes of cold ethanol, washed twice with 80% ethanol, dried, and redissolved in 30 μl of the assembly buffer. A freshly cleaved muscovite mica surface (Tedd Pella, Inc.) was treated with 20 mM NiCl_2_ for 2 min and washed with 100 μl of _dd_H2O. Purified triangles (2 nM final) were applied to the mica and incubated for 10 min, washed with 100 μl of _dd_H2O, and dried under a stream of compressed air. Atomic force microscopy (AFM) imaging of the triangles was performed using a 5500 AFM (Keysight Technologies) in alternate contact mode and the images were recorded with a 2 Hz scanning rate using a PPP-NCHR-50 probe from NanoAndMore USA Corp.

### Statistical analysis

Data is presented as the mean ± standard error of the mean (SEM). Statistical analyses were performed using Student’s *t* test or two-way analysis of variance (ANOVA) with Dunnet’s post hoc test as appropriate using commercially available software (GraphPad Prism, GraphPad Software, La Jolla, CA). In all experiments, results were considered statistically significant when a *P* value of less than 0.05 was obtained.

## Results

### Microglia show upregulated RIG-I protein expression following bacterial infection

**Fig. 1:**
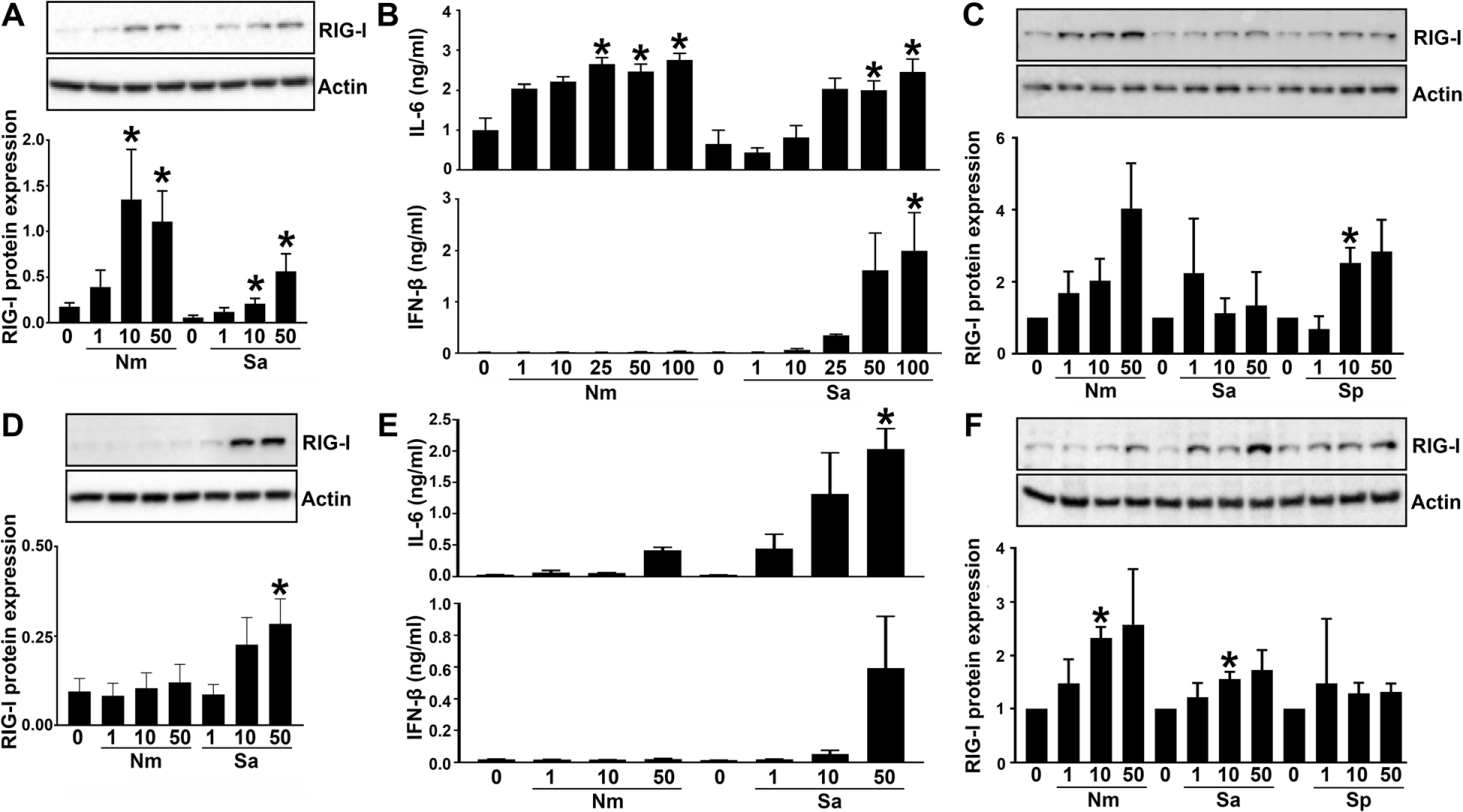
Bacterial infection stimulates cytokine release and increased RIG-I protein expression in glial cells. The human microglial cell line hμglia (**a**, **b**), primary murine microglia (**c**), primary human astrocytes (**d**, **e**), and primary murine astrocytes (**f**) were infected with *N*. *meningitidis* (Nm), *S*. *aureus* (Sa), or *S. pneumoniae* (Sp) at an MOI of 1, 10, or 50 (glial:bacteria). At 24 h post infection, cell lysates were collected and analyzed for RIG-I protein expression via immunoblot analysis (*N* = 3). RIG-I protein expression relative to that produced by unstimulated cells is shown in the corresponding bar graphs below each representative immunoblot (**a**, **c**, **d**, **f**). Cells were infected for 2 h with bacteria and at 24 h post infection, cell supernatants were collected and analyzed for cytokine production by specific capture ELISA for IL-6 and IFN-β (**b**, **e**). Data are expressed as mean ± SEM for a minimum of three independent experiments. Asterisks denote statistical significance compared to unchallenged cells as determined by Student’s *t* test (*p* < 0.05)

In order to establish the role of RIG-I in the detection of bacterial pathogens by glial cells, we first examined glial cell cytokine responses to bacterial infection. Consistent with our previous publications [1, 2, 39, 43, 44], we observed that primary human astrocyte and the hμglia human microglial cell line produce the inflammatory cytokine IL-6 (Fig. 1b, e) in response to infection with *N*. *meningitidis* and *S*. *aureus*. We also observed IFN-β production in response to bacterial infection 18 h post infection, but in contrast to IL-6, significant IFN-β production was only observed in response to infection with *S*. *aureus* indicating that release of interferons is pathogen specific. Importantly, we observed low constitutive expression of RIG-I in hμglia cells that was upregulated following infection with *N*. *meningitidis* or *S*. *aureus* (Fig. 1a). In contrast, RIG-I expression in primary human astrocytes was only upregulated following infection with *S*. *aureus* (Fig. 1d), indicating that such upregulation is both pathogen and cell type specific. The ability of bacterial pathogens to upregulate RIG-I expression was not restricted to human glia as low level constitutive expression of RIG-I in isolated primary murine astrocytes was significantly upregulated following bacterial infection with *N*. *meningitidis*, *S*. *aureus*, and *S*. *pneumoniae* while in primary murine microglia, we observed a trend for upregulated expression (Fig. 1c, f).

### Microglia show upregulated RIG-I protein expression in response to bacterial components

**Fig. 2:**
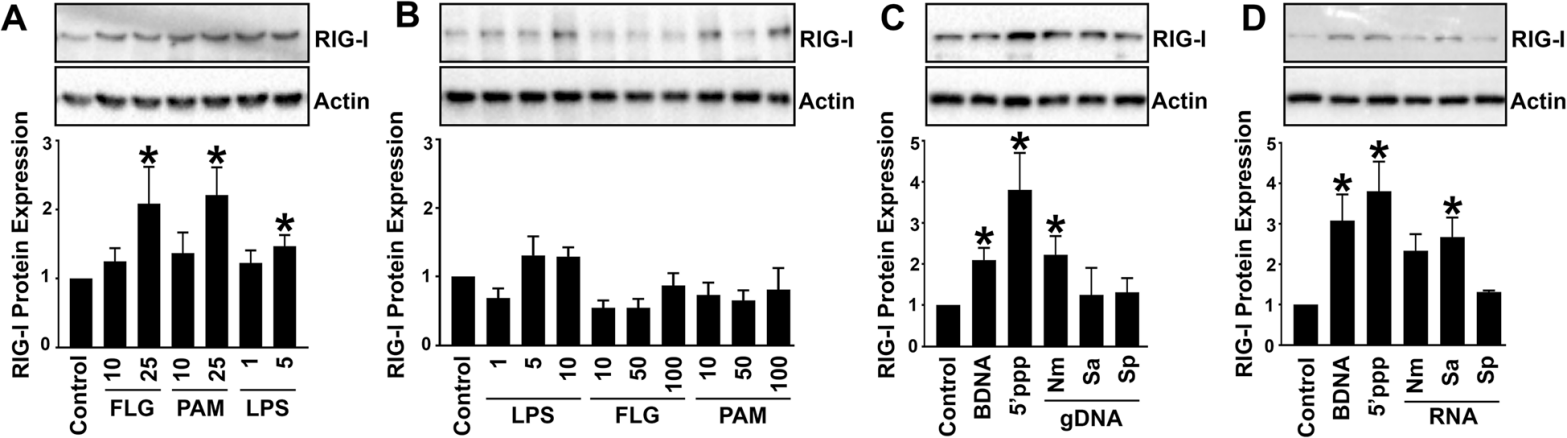
Bacterial components stimulate increased RIG-I protein expression in human microglia. Human microglial (**a**) and astrocytic (**b**) cell lines were stimulated with the bacterial components flagellin (FLG, 10–100 ng/ml), Pam3Cys (Pam, 10–100 ng/ml), and lipopolysaccharide (LPS, 1–10 ng/ml) for 18 h. In addition, hμglia human microglial cells were transfected for 4 h with 0.1 μg/ml BDNA, 1 μg/ml 5′pppRNA, 0.5 μg/ml bacterial genomic DNA (gDNA) (**c**) or 1 μg/ml bacterial RNA (**d**) and cell lysates were collected 24 h post transfection. Cell lysates were analyzed for protein expression of RIG-I and the housekeeping gene, β-actin, via immunoblot analysis. Relative RIG-I protein expression normalized to β-actin is displayed graphically below the representative immunoblot. Data are expressed as mean ± SEM for a minimum of three independent experiments. Asterisks denote statistical significance compared to unchallenged cells as determined by Student’s *t* test (*p* < 0.05).

To determine whether bacterial components are a sufficient stimulus for RIG-I induction, we challenged human astrocyte and microglia cell lines with PAM3Cys, LPS, and flagellin that are known specific ligands for TLR2, TLR4, and TLR5, respectively (Fig. 2a, b). Interestingly, hμglia cells showed significant upregulated RIG-I protein expression in response to these TLR ligands (Fig. 2a). In contrast, the human astrocyte cell line expressed low constitutive RIG-I expression, which was not elevated following stimulation with TLR ligands, even when challenged with higher doses (Fig. 2b).

We next examined if bacterial nucleic acids were sufficient to upregulate RIG-I expression. We found that transfection of hμglia cells with 5′-pppRNA, a RIG-I-specific ligand, significantly upregulated expression of its own receptor (Fig. 2c, d). Interestingly, we observed a similar upregulation in response to BDNA, a classic ligand for DNA sensors. It should be noted that it is known that the RNA sensor, RIG-I, can also recognize BDNA indirectly via RNA polymerase III-mediated conversion of BDNA to an RNA intermediate [19, 22]. Consistent with these findings, we observed that genomic DNA and RNA isolated from *N*. *meningitidis* and *S*. *aureus* upregulate expression of RIG-I by a human microglial cell line. Together, these data indicate that, in response to bacterial components, microglia show elevated expression of the cytosolic sensor RIG-I.

### Bacterial components stimulate IRF3 phosphorylation in human microglia

**Fig. 3:**
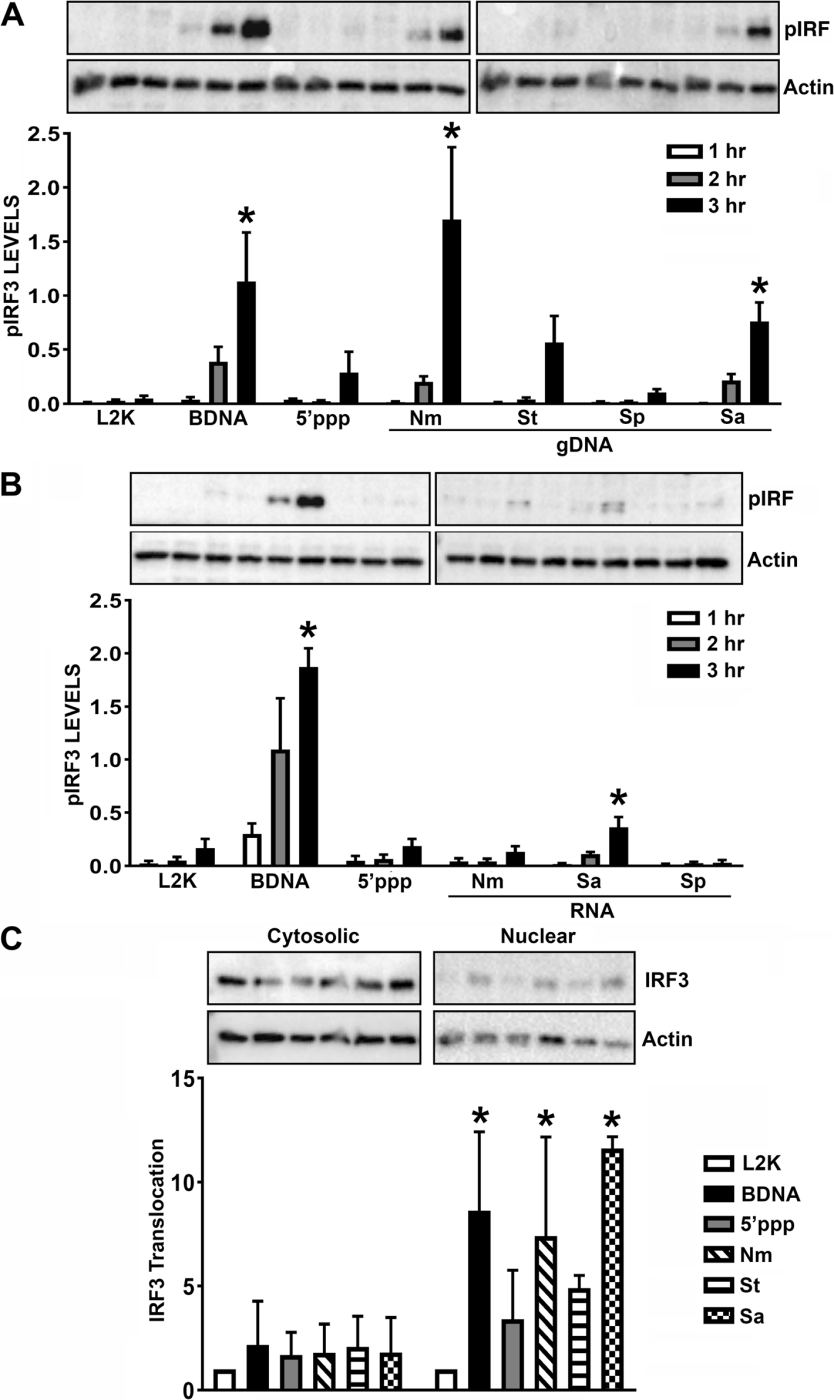
Bacterial nucleic acids stimulate IRF3 phosphorylation in hμglia human microglial cells. Cells were transfected with 0.1 μg/ml BDNA, 1 μg/ml 5′pppRNA, 0.5 μg/ml bacterial genomic DNA (gDNA) (**a**), or 1 μg/ml bacterial RNA (**b**). Cell lysates were collected at 1, 2, and 3 h and analyzed for protein expression of phosphorylated IRF3 (pIRF3) and the housekeeping gene, β-actin, via immunoblot analysis. Relative phosphorylated IRF3 normalized to β-actin is displayed graphically (**a**, **b**). Additionally, at 3 h post transfection, the cytosolic and nuclear cells fractions were separated and analyzed for protein expression of IRF3 and β-actin via immunoblot analysis (**c**). Relative IRF3 protein expression was normalized to β-actin and is displayed graphically below the representative immunoblot. Data are expressed as mean ± SEM for a minimum of three independent experiments. Asterisks indicate statistical significance compared to unchallenged cells as determined by two-way ANOVA (*p* < 0.05)

To further establish the functional role of RIG-I in glial cells following bacterial infection, we evaluated signaling downstream of RIG-I by assessing the level of IRF3 phosphorylation and nuclear translocation. As shown in Fig. 3, transfection of the human microglial cell line with BDNA induces IRF3 phosphorylation and nuclear translocation, as did 5′-pppRNA albeit to a more modest degree. Interestingly, transfection with genomic DNA or RNA isolated from *N*. *meningitidis*, *S*. *typhimurium*, *S*. *aureus*, or *S*. *pneumonia* induced IRF3 phosphorylation to varying degrees suggesting a bacterial species-specific difference in pathogen recognition by glial cells (Fig. 3a, b). Additionally, we observed that IRF3 phosphorylation occurred more rapidly in response to genomic DNA, with significantly more IRF3 phosphorylation compared to bacterial RNA at 3 h post transfection. Consistent with this observation, IRF3 nuclear translocation occurred at 3 h following administration of bacterial genomic DNA (Fig. 3c). Together, these data show that human microglial cells can sense bacterial genomic DNA and RNA leading to the induction of IRF3 phosphorylation and nuclear translocation.

### Bacterial nucleic acids stimulate RIG-I-dependent interferon responses in human microglia

**Fig. 4:**
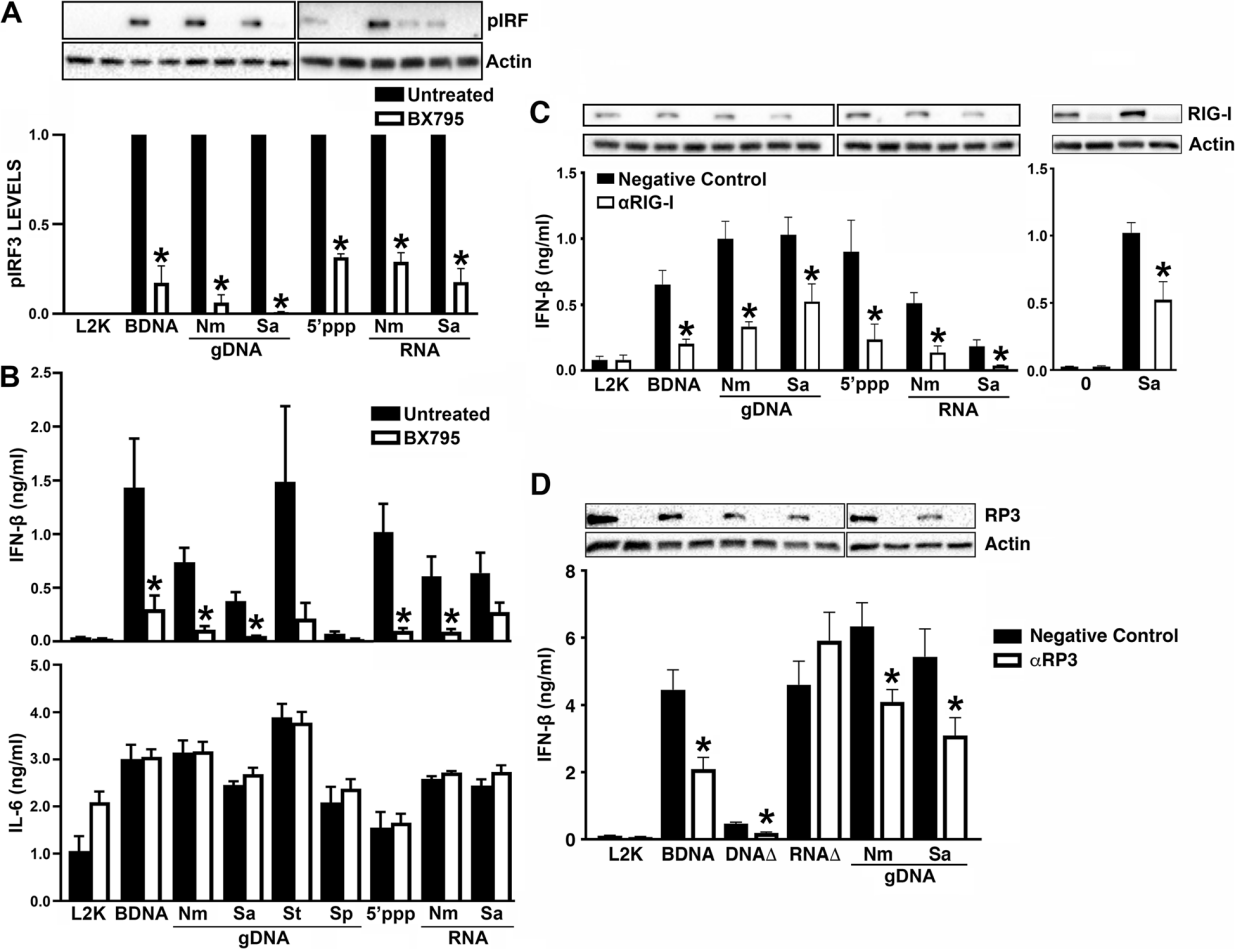
Bacterial components stimulate RIG-I-dependent signaling in hμglia human microglial cells. Cells were untreated or treated with 1 μM BX795, an inhibitor of TBK1/IKKε, for 3 h prior to transfection with 0.1 μg/ml BDNA, 1 μg/ml 5′pppRNA, 0.5 μg/ml bacterial genomic DNA (gDNA), or 1 μg/ml bacterial RNA (**a**, **b**). Total cell lysates were collected at 3 h and analyzed for protein expression of phosphorylated IRF3 (pIRF3) and β-actin via immunoblot analysis (**a**). Relative phosphorylated IRF3 expression was normalized to β-actin and is displayed graphically below the representative immunoblot. Cell supernatants were collected 24 h post transfection. IFN-β and IL-6 levels were quantified with specific capture ELISAs (**b**). Microglial cells were treated with scrambled siRNA or siRNA targeting RIG-I (αRIG-I) at a final concentration of 5 nM for 24 h. Cells were placed in fresh media for 24 h prior to transfection with 0.1 μg/ml BDNA, 1 μg/ml 5′pppRNA, 0.5 μg/ml bacterial gDNA, 1 μg/ml bacterial RNA, or infection with *S*. *aureus* at a MOI of 50 (**c**). Microglial cells were treated with scrambled siRNA or siRNA targeting RNA polymerase III (αRP3) at a final concentration of 5 nM for 24 h, then placed in fresh media for 24 h, and then transfected with 0.1 μg/ml BDNA, 0.5 μg/ml bacterial gDNA, or 5 nM DNA/RNA triangles (**d**). Cell supernatants were collected at 24 h post transfection and IFN-β levels were quantified by specific capture ELISA. Data are expressed as mean ± SEM for a minimum of three independent experiments. Asterisks indicate statistical significance compared to untreated condition as determined by Student’s *t* test or two-way ANOVA (*p* < 0.05)

To establish a role for RIG-I in microglial responses to either bacterial genomic DNA or RNA, we determined the effect of inhibiting TBK1 or the catalytic activity of IKK_Ɛ_ using the inhibitor, BX795. These signaling molecules are downstream of RIG-I ligand binding and are required for IRF3 phosphorylation and nuclear translocation. As shown in Fig. 4, BX795 treatment of hμglia human microglial cells diminished IRF3 phosphorylation in response to the control ligands BDNA and 5′-pppRNA. Similarly, BX795 treatment significantly reduces IRF3 phosphorylation in response to bacterial RNA (Fig. 4a). Interestingly, this inhibitor also significantly reduced levels of IRF3 phosphorylation in response to either *N*. *meningitidis* or *S*. *aureus* genomic DNA (Fig. 4a).

Consistent with these results, BX795 treatment significantly reduced microglial IFN responses to the control ligands, BDNA and 5′-pppRNA, and IFN-β production in cells stimulated with bacterial RNA. Furthermore, BX795 treatment also significantly reduced microglial IFN-β production in response to bacterial genomic DNA. In contrast to the effects on microglia type I IFN responses, inhibition of TBK1/IKK_Ɛ_ catalytic activity did not affect release of the inflammatory cytokine IL-6 (Fig. 4b), thus suggesting that an alternative signaling cascade underlies this inflammatory cytokine response.

Finally, in order to confirm that bacterial components stimulate RIG-I-mediated responses, expression of this cytosolic sensor was knocked down using an siRNA approach in microglia prior to challenge with DNA and RNA ligands. As shown in Fig. 4c, RIG-I knockdown significantly reduced hμglia IFN responses to RNA ligands including 5′-pppRNA, *N*. *meningitidis* RNA, and *S*. *aureus* RNA. In addition, we observed a reduction in IFN responses to DNA ligands including BDNA, *N*. *meningitidis* DNA, and *S*. *aureus* DNA. Importantly, the physiological relevance of such RIG-I-mediated microglial responses is demonstrated by the ability of RIG-I knockdown to significantly reduce the production of IFNs by hμglia cells following *S*. *aureus* infection (Fig. 4c)*.* Together, these data indicate that RIG-I contributes to microglial IFN responses to bacterial infection and not only serves as a receptor for bacterial RNA but also contributes to microglial responses to bacterial genomic DNA.

RIG-I is known to identify DNA ligands indirectly via the activity of RNA polymerase III [19, 22]. In order to confirm that RIG-I-mediated detection of bacterial genomic DNA by human microglial cells is dependent on RNA polymerase III, expression of the catalytic RNA polymerase III subunit A was knocked down using an siRNA approach prior to challenge with DNA ligands. As shown in Fig. 4d, RNA polymerase III knockdown significantly reduced hμglia cell IFN responses to the DNA ligands, BDNA, *N*. *meningitidis* DNA, and *S*. *aureus* DNA, indicating that RIG-I-mediated detection of DNA ligands in human microglial cells occurs in an RNA polymerase III-dependent manner.

### Nucleic acid nanoparticles stimulate RIG-I -dependent responses in human microglia

Within the CNS, damaging proinflammatory responses are initiated by resident microglia [1, 2] and we have demonstrated that RIG-I is upregulated in response to bacterial components, which in part mediates the production of type I IFNs. As such, RIG-I may be a targetable receptor to promote IFN responses within the CNS. We have previously demonstrated that RNA polygons stimulate microglia to release IFN-β [33, 45] and in the present study we have determined whether RIG-I underlies such responses.

**Fig. 5:**
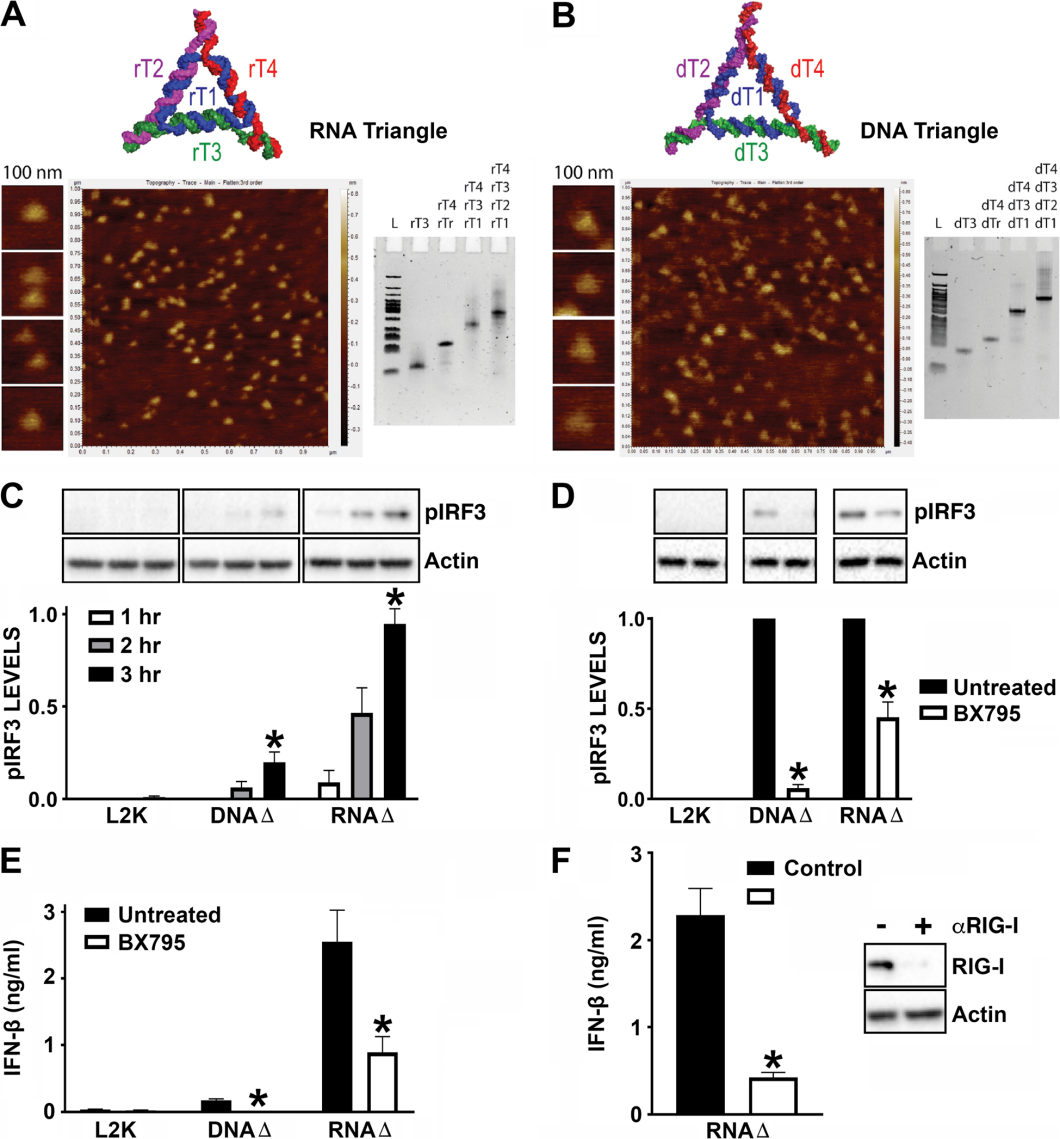
RNA triangle nanoparticles stimulate RIG-I dependent responses in hμglia human microglial cells. DNA and RNA triangle formations assessed by atomic force microscopy (AFM) and ethidium bromide total staining native-PAGE (**a**, **b**). “L” corresponds to DNA low molecular weight ladder. Cells were untreated or treated with 1 μM BX795, an inhibitor for TBK1/IKKε, for 3 h prior to transfection with 0.1 μg/ml BDNA, 1 μg/ml 5′pppRNA, 5 nM DNA triangles, or 5 nM RNA triangles. Cell lysates collected at 1, 2, or 3 h were analyzed for protein expression of phosphorylated IRF3 (pIRF3) and the housekeeping gene β-actin via immunoblot analysis (**c**, **d**). Cell supernatants were collected 24 h post transfection and IFN-β levels were quantified by specific capture ELISA (**e**). Microglia were treated with scrambled control siRNA or siRNA targeted against RIG-I (αRIG-I) at a final concentration of 5 nM for 24 h. Cells were placed in fresh media for 24 h prior to transfection with 5 nM RNA triangles. Cell supernatants were collected 24 h post transfection and IFN-β levels were quantified by specific capture ELISA. Cell lysates were collected 24 h post transfection and analyzed for protein expression of RIG-I and the housekeeping gene, β-actin, via immunoblot analysis (**f**). Data are expressed as mean ± SEM for a minimum of three independent experiments. Asterisks indicate statistical significance compared to untreated condition as determined by Student’s *t* test or two-way ANOVA (*p* < 0.05)

As shown in Fig. 5a, b, we visualized fully assembled RNA and DNA triangles by AFM and the native-PAGE experiments confirmed the migration retardation of fully assembled nucleic acid-based nanoparticles (NANPs) compared to their partial assemblies (monomer, dimer, and trimer). The assembly yields, estimated based on native-PAGE analysis, were greater than 90% for both NANPs. We then evaluated IRF3 phosphorylation in hμglia microglial cells in response to NANPs transfection, and found that RNA NANPs stimulate rapid responses as early as 2 h following transfection (Fig. 5c). Importantly, BX795 treatment significantly reduced NANPs’ stimulated IRF3 phosphorylation and interferon production (Fig. 5d, e).

The RNA strands of RNA triangle NANPs were produced by in vitro run-off transcription [33, 46] thus making all four strands in NANPs’ composition possess 5′-triphosphate groups, a reported ligand for RIG-I recognition [18, 21, 47]. In order to verify if RIG-I is required for microglial responses to RNA triangle NANPs, RIG-I expression was knocked down using siRNA prior to transfection of hμglia cells with RNA NANPs. As shown in Fig. 5f, IFN production was significantly reduced following RIG-I knockdown indicating that these RNA nanoparticles can serve as a RIG-I agonist (Fig. 5f). Furthermore, in agreement with the results obtained using bacterial genomic DNA, RNA polymerase III knockdown significantly reduced microglial IFN responses to DNA NANPs, indicating that RIG-I-mediated detection of DNA NANPs also occurs via an RNA polymerase III-dependent mechanism (Fig. 4d).

## Discussion

It is now appreciated that resident CNS cells, microglia and astrocytes, express PRRs to identify pathogen motifs and are critical for shaping the immune response to infection [1, 2, 6–9, 14]. We have previously demonstrated that RIG-I expression in murine microglia and astrocytes and human astrocytes is inducible following challenge with vesicular stomatitis virus (VSV) or herpes simplex virus-1 (HSV-1), neurotropic RNA and DNA viruses, respectively [3, 9, 14]. Consistent with our previous findings, we observed constitutive expression of RIG-I in murine microglia and human astrocytes, but here, we also provide the first demonstration of RIG-I protein expression in human microglial cells. Interestingly, we determined that RIG-I expression can be further elevated in human glia in response to bacterial infection, and our data indicates that this effect is pathogen and cell type specific as human astrocytes only show induced expression in response to the Gram-positive pathogen, *S*. *aureus*. Furthermore, we have identified bacterial motifs that are recognized by either surface PRRs or cytosolic PRRs that are sufficient to induce RIG-I protein expression in microglial cells. Again, we found cell type-dependent differences as human astrocytes did not show increased expression of RIG-I following stimulation with ligands for surface TLRs. However, it is presently unclear whether the ability of bacteria and their ligands to upregulate RIG-I expression in human microglial cells but not astrocytes occur as a direct effect of these stimuli or, rather, occurs secondary to the production of other mediators. Furthermore, it will be interesting to determine whether RIG-I-specific ligands can upregulate the expression of other PRRs, thereby sensitizing human microglia to bacterial challenge in a similar “crosstalk” manner to that previously shown for other PAMPs in glia [6–9, 48].

While RIG-I has classically been defined as a viral pattern recognition receptor, more recent studies have indicated that RIG-I can serve a role in the detection of bacterial pathogens in peripheral cells types [22, 26–31]. The present study indicates that RIG-I can serve directly as a sensor for pathogen RNA and indirectly as a sensor for pathogen DNA in an RNA polymerase III-dependent mechanism. Interestingly, the ability of RIG-I to serve as a sensor for pathogen RNA versus DNA appears to be both pathogen and glial cell type specific. As anticipated, we showed RIG-I-dependent responses to the RIG-I-specific ligand, 5′-pppRNA, but we also demonstrated that this sensor was responsible, at least in part, for glial responses to BDNA, that can be converted by RNA polymerase III to a ligand for RIG-I. Importantly, in agreement with studies in peripheral cell types, we have demonstrated that RIG-I can identify bacterial RNA in glial cells. More interestingly, our data supports a model in which RIG-I also serves indirectly as a sensor for bacterial genomic DNA, and does so in a bacterial species-dependent manner. A possible explanation for this observation may come from the previous demonstration that RIG-I has sequence preferences for polyuridine-rich motifs or polyuridine motifs that are interspersed with cysteine nucleotides [20, 49]. Additionally, RNA polymerase III preferentially recognizes AT-rich DNA [19, 22]. As such, differences in bacterial genome characteristics and architecture may account for varying RIG-I-mediated glial responses between bacterial species.

Our previously published studies have demonstrated that nucleic acids can serve as building blocks for the construction of NANPs [33, 34, 46, 50]. We have shown that the RNA and DNA composition of such assemblies dictate their molecular weight, melting temperature, and half-life, and quantitative structure-activity relationship models indicate that these properties strongly predict NANPs immunostimulatory activity [45]. We have demonstrated that RNA triangle NANPs are potent inducers of type I IFN in human microglia [33, 45], while DNA triangle NANPs, albeit to a lesser degree, can also induce demonstrable production of type I IFN. In the present study, we determined whether RIG-I mediates glial IFN responses to RNA or DNA triangles. Since RNA strands synthesized by run-off transcription of a DNA template are the building blocks for RNA triangle nanoparticles and contain 5′-triphosphate groups, a known ligand motif for RIG-I [18, 21, 47], it was not surprising that RNA triangles elicited IFN production in a RIG-I-dependent manner. Interestingly, we determined that glial cell responses to DNA triangles are also dependent on RIG-I signaling pathways due to an RNA polymerase III-dependent mechanism.

## Conclusions

In the present study, we have demonstrated a human microglial cell line and primary human astrocytes constitutively express RIG-I, and we have shown that such expression is elevated following bacterial infection. Known ligands for membrane-bound TLRs and bacterial nucleic acids are also capable of inducing RIG-I expression in a human microglial cell line. Importantly, bacterial nucleic acids stimulate RIG-I-dependent signaling and IFN production by human microglial cells, and our data demonstrates that nucleic acid nanoparticles can serve as agonists of RIG-I and stimulate RIG-I-dependent signaling and IFN production by these cells. This raises the exciting prospect of RIG-I as a druggable target, and rationally designed nucleic acid nanoparticles may serve as a platform for targeting this immune receptor. In terms of pathogen infection within the CNS, such RIG-I agonists could enhance IFN responses that may be protective in contrast to the damaging proinflammatory responses initiated by glial cells during infection. Furthermore, RIG-I has previously been identified as a candidate target for antivirals, vaccine adjuvants, and antitumor agents [51–54]. Therefore, further exploration of RIG-I activation and signaling in glia will provide the necessary knowledge for designing nanoscaffolds tailored to initiate desired immune responses for a broad range of therapeutic applications.

## Supplementary information

**Additional file 1:.** Supplemental 1

## Data Availability

The data used and/or analyzed during the current study available from the corresponding author on reasonable request.

## Abbreviations

ATCC: American type culture collection
ANOVA: Analysis of variance
CNS: Central nervous system
CFU: Colony forming units
CSF-1: Colony-stimulating factor-1
DMEM: Dulbecco’s modified Eagle’s medium
EMEM: Eagle minimum essential media
FBS: Fetal bovine serum
FLG: Flagellin
GFAP: Glial fibrillary acidic protein
HSV-1: Herpes simplex virus-1
HRP: Horseradish peroxidase
IKKε: IkappaB kinase-ε
IFN: Interferon
IRF3: Interferon regulatory factor 3
ISGs: Interferon-stimulated genes
L2K: Lipofectamine 2000
LPS: Lipopolysaccharide
*L. pneumophila*: *Legionella pneumophila*
*L. monocytogenes*: *Listeria monocytogenes*
LB: Lysogeny broth
MOI: Multiplicities of infection
*N. meningitidis*, Nm: *Neisseria meningitidis*
NLR: NOD-like receptors
NANPs: Nucleic acid-based nanoparticles
NOD: Nucleotide oligomerization domain
Pam3Cys: Pam3Cys-Ser-(Lys)4
PRR: Pattern recognition receptors
RIG-I: Retinoic acid inducible gene I
RLRs: Retinoic acid inducible genes (RIG)-I-like receptors
*S. enterica Serovar typhimurium*, *St*: *Salmonella enterica Serovar Typhimurium*
αRIG-I: siRNA targeted against RIG-I
*S. aureus*, Sa: *Staphylococcus aureus*
*S. pneumoniae*, Sp: *Streptococcus pneumoniae*
TBK1: TANK-binding kinase 1
TLRs: Toll-like receptors
VSV: Vesicular stomatitis virus
