# Supplementary material for: Retinoic acid inducible gene-I mediated detection of bacterial nucleic acids in human microglial cells
Source: J Neuroinflammation. 2020 May 1;17:139. doi: 10.1186/s12974-020-01817-1 (PMC7195775; doi:10.1186/s12974-020-01817-1)
Supplement: Supplementary file 1 — Additional file 1:. Supplemental 1 [file 12974_2020_1817_MOESM1_ESM.pdf]

SUPPLEMENTAL 1: Sequences used for RNA and DNA triangles:

Triangles were assembled by combining equimolar solutions of the following strands:

DNA Triangle:

dT_1

5`-

GGATGCTGGTACTTTTGAAACATTTCGAGTCGCGAGGGTTTTCCCATCGTTGGCCCG

TA

TCGCGTTTTCTTATGAAGA

dT_2

5`-GGTCGCGACCTTCTTTTCCCTCGCGACTCGAAATGTTTCTTTTCGAGGTCGCCC

dT_3

5`-GGATCTTTCGCCTTTTCGCGATACGGGCCAACGATGGGTTTTGAAGGTCGCGAC

dT_4

5`-GGGCGACCTCGTTTTGTACCAGCATCCTCTTCATAAGTTTTGGCGAAAGATCC

RNA Triangle:

rT_1

5`-

GGAUGCUGGUACUUUUGAAACAUUUCGAGUCGCGAGGGUUUUCCCAUCGUUGGC

CC

GUAUCGCGUUUUCUUAUGAAGA

rT_2
